# Relation between the first psychotic episode in schizophrenia patients and IL-1β plasma levels – Serbian population study

**DOI:** 10.1192/j.eurpsy.2024.798

**Published:** 2024-08-27

**Authors:** N. M. Stojanovic, S. Tošić Golubović, S. Vujić, N. Stefanović, G. Nikolić, A. Todorović, A. Vrućinić, M. Petković, M. Simonović, P. Randjelovic, T. Jevtović Stoimenov

**Affiliations:** ^1^Department of Physiology; ^2^Department of Psychiatry, Faculty of Medicine, University of Niš; ^3^Clinic for Psychiatry, University Clinical Centre; ^4^Faculty of Medicine, University of Niš; ^5^Centre for mental health, University Clinical Centre; ^6^ Special Psychiatric Hospital; ^7^Department of Psychiatry, University Clinical Centre; ^8^Department of Biochemistry, Faculty of Medicine, University of Niš, Niš, Serbia

## Abstract

**Introduction:**

According to immunological theories of schizophrenia prenatal and postnatal exposure to pathogens may contribute to the etiopathogenesis, suggesting that chronically activated immune system cells (macrophages and T lymphocytes) constantly secrete proinflammatory cytokines which affect the development and function of central nervous system.

**Objectives:**

In the present work we aimed to evaluate IL-1β plasma levels in schizophrenic patients during their first psychotic episode and to compare the obtained results to those from healthy subjects.

**Methods:**

Plasma was obtained from 32 drug-naive schizophrenic patients, without history of substance abuse or addiction, immediately after their admission to the medical ward, while the control samples were obtained from 20 healthy volunteers.

**Results:**

Levels of IL-1β were measured using ELISA assay, which measures IL-1β protein in a range from 7.81 to 500 pg/ml. Results revealed that the levels of IL-1β in patients with first psychotic episode were not increased and were below the limit of detection in all studied samples. The same was found in the samples belonging to the control group.

**Conclusions:**

These data contribute to the poll of knowledge and a still unresolved dogma about the etiopathogenesis of schizophrenia since the results obtained by some studies are also questioning this marker. Thus, whether or not an increase of IL-1β is congenital, acquired during the prodromal phase or absent until the time of first psychotic episode has not yet been investigated.

**Disclosure of Interest:**

None Declared

